# The Altered Triple Networks Interaction in Depression under Resting State Based on Graph Theory

**DOI:** 10.1155/2015/386326

**Published:** 2015-06-09

**Authors:** Hongna Zheng, Lele Xu, Fufang Xie, Xiaojuan Guo, Jiacai Zhang, Li Yao, Xia Wu

**Affiliations:** ^1^College of Information Science and Technology, Beijing Normal University, No. 19 Xin Jie Kou Wai Da Jie, Beijing 100875, China; ^2^State Key Laboratory of Cognitive Neuroscience and Learning and IDG/McGovern Institute for Brain Research, Beijing Normal University, Beijing 100875, China; ^3^Center for Collaboration and Innovation in Brain and Learning Sciences, Beijing Normal University, Beijing 100875, China; ^4^State Key Laboratories of Transducer Technology, Chinese Academy of Sciences, Shanghai 200050, China

## Abstract

The triple network model (Menon, 2011) has been proposed, which helps with finding a common framework for understanding the dysfunction in core neurocognitive network across multiple disorders. The alteration of the triple networks in the major depression disorder (MDD) is not clear. In our study, the altered interaction of the triple networks, which include default model network (DMN), central executive network (CEN), and salience network (SN), was examined in the MDD by graph theory method. The results showed that the connectivity degree of right anterior insula (rAI) significantly increased in MDD compared with healthy control (HC), and the connectivity degree between DMN and CEN significantly decreased in MDD. These results not only supported the proposal of the triple network model, but also prompted us to understand the dysfunction of neural mechanism in MDD.

## 1. Introduction

Human brain is a complex neural network; many psychological and neurological disorders are associated with the dysfunction of multiple brain regions or networks [1–4]. Based on that, Menon proposed a triple network model which helps in finding a common framework for understanding cognitive and affection disorders [4]. Major depression disorder (MDD), as one of the main kinds of affection disorders, is characterized by stable, pervasive depressive mood, guilt, disinterest, worthlessness, and even tendency of suicide [5]. It seriously impacts the daily lives of patients as well as their family and also brings about significant economic and professional functioning burdens to society [6]. It is important to investigate the altered interaction of the triple networks in MDD, which will help to understand the neural mechanism of MDD.

Three networks, default model network (DMN), central executive network (CEN), and the salience network (SN), are included in the triple network model. DMN decreases neural activity when performing task and increases activity in the resting [1]; CEN is responsible for high-level cognitive functions and external information procession [4] and the SN keeps homeostatic interoception and external stimulus [7]. In particular, anterior insula (AI) within SN is a hub of the large scale brain networks and is applied to accommodate the dynamic interaction between the internal self-perception and external orient stimulus [4, 8]. In recent years, dysfunction of the three cognitive networks has remarkably occurred in many mental and neurological disorders [2, 9–12]. For instance, the intraintrinsic functional connectivity (intra-iFC) was altered in patients' DMN, CEN, and SN and the interintrinsic functional connectivity (inter-iFC) between the SN and CEN was increased in schizophrenia [12]. All of these studies suggested that the triple network model may offer a new insight to understand the brain mechanism of MDD.

The study of Sheline et al. (2010, PNAS) in MDD was to explore the altered connectivity between the dorsal medial prefrontal cortex region and the three important networks: the cognitive control networks, default mode network, and affective network. They determined significant differences using the three a priori seed regions by mean correlation coefficients between the MDD and HC [10]. In Manoliu et al.'s study the participants' spatial maps were compared by two-sample *t*-test among the interested networks (anterior DMN, inferior posterior DMN, superior posterior DMN, SN, left ventral CEN, right ventral CEN, and dorsal CEN); patient with MDD showed decreased intra-iFC within SN right AI. The decreased inter-iFC between the DMN and CEN and increased inter-iFC between the SN and DMN after the subjects' specific time courses were used to analyze the inter-iFC among these interested networks [13]. Furthermore, many studies based on functional magnetic resonance imaging (fMRI) have reported that the abnormality of the functional connectivity in the intrinsic brain neural mechanism contributes to the MDD [14]. Yuen et al.'s study showed that resting state functional connectivity between the right anterior insula (rAI) and right posterior parietal cortex (rPPC) increased in the apathy of late-life depression [15]; the research of Lemogne et al. indicated that patients in depression displayed an increasing functional connectivity between the medial frontal gyrus (MFG), dorsal anterior cingulate cortex (dACC), and the dorsolateral prefrontal cortex (dlPFC) [16], and Strigo et al. reported that the depression subjects showed lower rAI activity related to anticipatory shift in stimulus intensity [17]. However, the interaction among networks of the nodes in the triple networks of MDD is not clear.

In this study, functional connectivity of the nodes and the interaction between the triple networks (DMN, CEN, and SN) in MDD and HC under the resting state fMRI were examined by graph theory method, which has been applied to the multiple brain regions functional connectivity in both the resting state and the motor task [18].

## 2. Materials and Methods

### 2.1. Participants

Sixteen MDD (four males, mean age 33.13 years) and sixteen age-, sex-, and education-matched HC participated in this study. The MDD were recruited from the Anding Hospital, Capital Medical University, while the participants in the HC group were recruited through newspaper advertisements. All the subjects in MDD met the American Psychiatric Association DSM-IV diagnostic criteria of depression, and HC were interviewed using the Structured Clinical Interview for DSM-IV, nonpatient edition. Before experiment, all of the subjects wrote informed consent by themselves. The clinical characteristics of MDD and HC were shown in Table 1.

### 2.2. fMRI Data Acquisition

All the resting state functional images scans were acquired on a 3.0-Tesla scanner (Siemens, Erlangen, Germany) in the National Key Laboratory for Cognitive Neuroscience and Learning, Beijing Normal University, using a single-shot T2^*^ weighted gradient echo-planar imaging (EPI) sequence, with the following parameters: repetition time (TR) = 2000 ms, echo time (TE) = 30 ms, flip angle (FA) = 90°, matrix size = 64 × 64, field of view (FOV) = 220 mm × 220 mm, total 240 volumes, slice thickness = 3.5 mm, skip = 0.6 mm, and slices number 33. All participants were kept in resting state, remained quiet, without moving, eyes closed, no sleeping, and no system thinking activities during functional MRI scanning.

### 2.3. Data Preprocessing

Firstly, the data preprocessing was performed based on the software of SPM8 (statistical Parametric Mapping 8, http://www.fil.ion.ucl.ac.uk/spm). Each fMRI scan contained a total of 240 times points; the first 10 volumes were discarded due to signal stabilization and subjects' adaptation to the scanner's noise.

After that, slicing timing and realignment, spatially normalized into standard stereotaxic space and smoothing image volumes with an 8 × 8 × 8 full-width at half maximum (FWHM) Gaussian kernel, were performed.

Finally, the images of all subjects were done with detrend in order to remove linear trend, filtered in the bandwidth of 0.01~0.08 Hz to reduce the high-frequency interference with the Resting State fMRI Data Analysis Toolkit (REST, http://resting-fmri.sourceforge.net).

### 2.4. Defining the Nodes

Independent component analysis (ICA) has been reported as an appropriate method to explore the fMRI data in functional connectivity analysis [19] and has been well used in the resting state fMRI analysis [20]. In this study we used the group ICA of fMRI toolbox (GroupICAT v2.oc, http://icatb.sourceforge.net/) to obtain the brain spatial pattern maps.

In order to make sure the same components were identified in each subject, group ICA treated all subjects as one group [21]. In the current study, 22 components were chosen according to the minimum description length (MDL) method. Furthermore, the components of DMN, CEN, and SN were selected according to the previous studies about the triple networks through visual inspection [4]. After that, the spatial patterns of DMN, CEN, and SN were generated by one sample *t*-test with the software of SPM8.

In our study, the nodes were defined based on the spatial pattern maps. As reported in previous studies, the critical brain regions for DMN are posterior cingulate cortex (PCC), ventral medial prefrontal cortex (vmPFC), and angular gyrus (Ang) [22]. CEN mainly includes the dorsolateral prefrontal cortex (dlPFC) and posterior parietal cortex (PPC) [23]. SN consists of dorsolateral anterior cingulate cortex (dACC) and anterior insula (AI) [7]. The coordinates were determined according to the highest *T*-value in the spatial pattern maps shown in Table 2 in detail. After that, we defined spheres with radius of 3 mm for each node as the masks, centered on the coordinates of each node determined. Then, the associated average time series of each node was extracted for each subject.

### 2.5. Functional Connectivity Analyses

For the graph theory method, the nodes are denoted by nodes in a graph and the links between the nodes represent the functional connectivity between them. The interregional connectivity degree between nodes *i* and *j* was defined as

(1) $$\begin{array}{c} \eta_{ij}=e^{-\xi d_{ij}}, \end{array}$$

where *ξ* is a positive real constant; here we set it equal to 2 and it indicates how the interregional connectivity changes with the distance between the two nodes [18]. *d*_*ij*_ denotes the distance between nodes *i* and *j* calculated by Golay et al. 1998 [24], defined as

(2) $$\begin{array}{c} d_{ij}=\frac{\left(1-c_{ij}\right)}{\left(1+c_{ij}\right)}, \end{array}$$

where *c*_*ij*_ represents the Pearson correlation coefficient between the two time series of nodes *i* and *j*:

(3) $$\begin{array}{c} c_{ij}=\frac{\mathrm{cov}\left(i,j\right)}{\sigma_{i}\sigma_{j}}. \end{array}$$

The larger the value of *η* means the closer the interaction between the two brain regions.

In this study, in order to explore the dynamic interaction between the three networks of the patients with depression in rest we defined two formulas with the graph theory method. One is for the intrinsic functional connectivity of all the nodes included in the three networks (DMN, CEN, and SN). The other is for the investigation of internetwork intrinsic functional connectivity between the three cognitive networks of DMN, CEN, and SN, which help to further understand the relationship between the three networks. The specific formulas are as follows.

#### 2.5.1. The Degree of the Node

We define the sum of all the functional interregional connectivities *η* between *i* and all other nodes in the three networks

(4) $$\begin{array}{c} \Gamma_{i}=\overset{n}{\underset{j=1}{\sum }}\eta_{ij} \end{array}$$

as the connectivity degree of the node *i* in a graph. In this study, during the graph theory calculation, we put all the 11 nodes in one graph to obtain the degree of each node. This means that the larger the degree of node *i* the more the importance of node *i* in the graph, which also means that the greater the impact of node *i* on other brain regions in the network. Then we normalized the Γ_*i*_:

(5) $$\begin{array}{c} \overset{-}{\Gamma_{i}}=\frac{\Gamma_{i}}{\sum_{j=1}^{n}\Gamma_{j}}; \end{array}$$

in this study the $\bar{\Gamma_{i}}$ presents the functional connectivity of the node *i* with the other nodes in the three networks. For each node the alteration of $\overset{-}{\Gamma }$ between the two groups was tested using two-sample *t*-test.

Moreover, it is necessary to investigate the significantly altered interregional connectivity that related to the node *i*. Thus, the nodes were significantly altered in $\overset{-}{\Gamma }$; further exploration on the interregional connectivity *η* between two nodes was carried out. In particular, for each node *i*, the interregional connectivity *η*_*ij*_ = *e* ^−*ξd*_*ij*_^ for each *j* ≠ *i* was analyzed statistically by two-sample *t*-test between depression and HC.

#### 2.5.2. Connectivity Degree of the Network

In order to further explore the functional connectivity between the three networks, we defined a calculation for the network analysis as follows:

(6) $$\begin{array}{c} \Gamma_{MN}=\overset{n}{\underset{i=1}{\sum }}\overset{m}{\underset{j=1}{\sum }}\eta_{ij}, \end{array}$$

where Γ_*MN*_ is the connectivity degree between the two networks *M* and* N*;* m* and *n* denote the number of nodes in the networks *M* and *N*, respectively; *η*_*ij*_ denotes the interregional connectivity between nodes *i* and *j*, defined as *η*_*ij*_ = *e* ^−*ξd*_*ij*_^; the *i* represents the node of the network *M*; and the *j* is the node in the network *N*. The larger the value of Γ_*MN*_, the closer the functional connectivity between the two networks. The two-sample* t*-test was used to analyze the alteration of Γ_*MN*_ for each network between the two group subjects.

## 3. Result

### 3.1. Spatial Pattern of the Triple Networks

**Figure 1:**
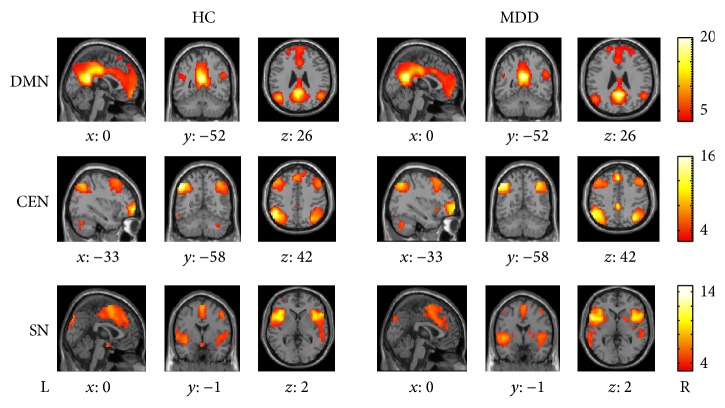
The DMN, CEN, and SN spatial pattern maps of HC and MDD. The statistical maps displaying one sample *t*-test of the two groups, with the color scale representing the ranges of *t*-values.

In our study the nodes of DMN were PCC, vmPFC, right Ang (rAng), and left Ang (lAng). CEN mainly included the right dlPFC (rdlPFC), left dlPFC (ldlPFC), right (rPPC), and left PPC (lPPC). SN consisted of dACC, right AI (rAI), and left AI (lAI). The group spatial pattern maps of the DMN, CEN, and SN were shown in Figure 1. The coordinates determined according to the highest *T*-value were shown in Table 2.

### 3.2. Alteration of Connectivity Degree of Nodes

**Figure 2:**
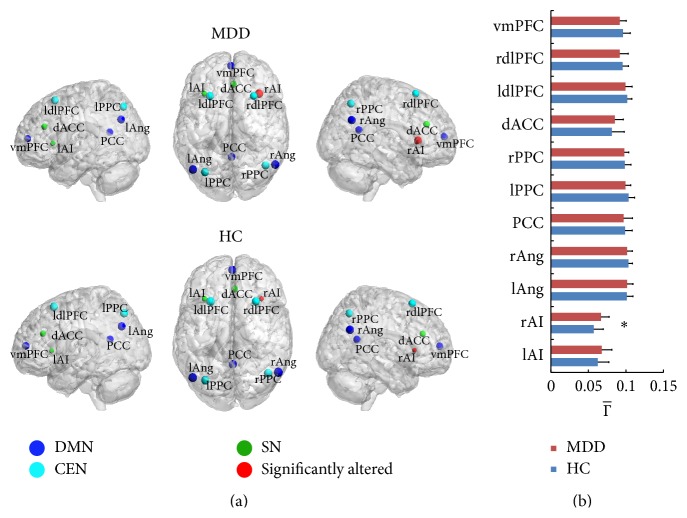
The region connectivity degree of MDD and HC. In (a) the size of the node visualizes the value of the connectivity degree $\overset{-}{\Gamma }$ of all the 11 nodes. Red dot indicates the nodes that significantly altered for the MDD compared to HC; the other colored dots mean “not significant.” The blue dots are the nodes in DMN and dark green dots in CEN; bright green dots indicate the nodes in SN. (b) shows the $\overset{-}{\Gamma }$ of all the 11 nodes (∗ represents significance with *P* < 0.05).

After the two-sample* t*-test for each node between MDD and HC, significant increase in the connectivity degree of the rAI was detected in MDD (*P* < 0.05). The alteration also existed in other brain regions though not significant statistically. For MDD, the mean connectivity degree of lAI and dACC increased and the mean degree for vmPFC, PCC, bilateral angular gyrus (rAng and lAng), bilateral dlPFC (rdlPFC and rdlPFC), and bilateral PPC (rPPC and lPPC) decreased compared with HC (Figure 2).

### 3.3. Alterations of Interregional Connectivity between Nodes

**Figure 3:**
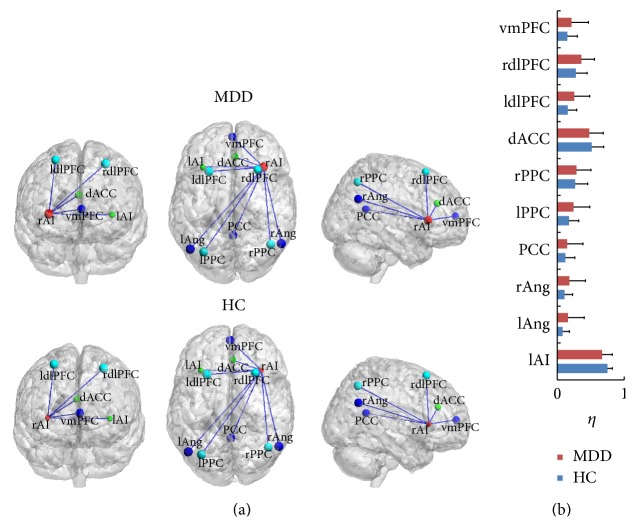
The interregional connection *η* between rAI and other brain regions in MDD and HC. (a) The lines showed the visualization of *η*. (b) shows the value *η* between the rAI and all the other nodes.

The degree of rAI significantly increased in MDD compared to the HC just as above shown (Figure 2). Therefore the interregional connectivity (*η*) between the rAI and other nodes was measured in further analysis. For MDD, the *η* between rAI and vmPFC, rdlPFC, ldlPFC, rPPC, lPPC, PCC, rAng, and lAng was larger than HC although the alteration was not significant after the statistical analysis by two-sample *t*-test. The result is shown in Figure 3.

### 3.4. Alterations of Connectivity Degree between Networks

**Figure 4:**
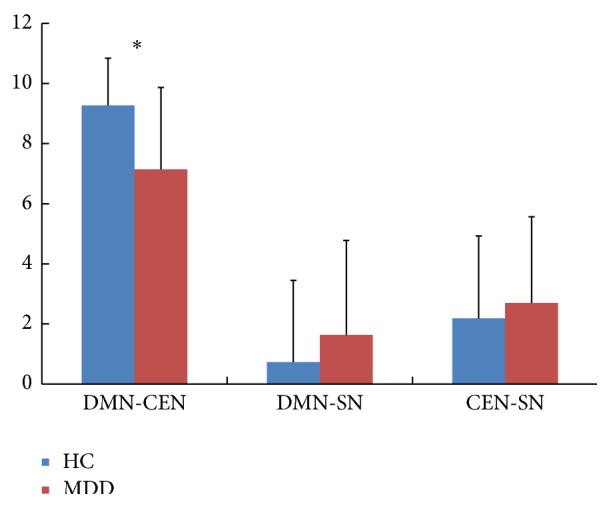
The connectivity degree Γ between the networks. Γ is the functional connectivity degree between the networks for the two groups (∗ represents significance by two-sample *t*-test).

After the investigation of the connectivity degree between networks with Γ_*MN*_ = ∑_*i*=1_^*n*^∑_*j*=1_^*m*^ *η*_*ij*_ and two-sample *t*-test, the result showed that the connectivity degree between the DMN and CEN significantly decreased in MDD (two-sample *t*-test, *P* < 0.05). The interaction degree of the networks DMN and SN, CEN, and SN increased in MDD. The details are shown in Table 3 and Figure 4.

## 4. Discussion

To investigate the relationship among the important cognitive and affective related brain regions of the triple networks (DMN, CEN, and SN) in MDD, the functional connectivity of the nodes and the altered interaction of the triple networks (DMN, CEN, and SN) were examined by using the graph theory method in the resting state fMRI. The results demonstrated the significantly increased degree of rAI in MDD and the significantly decreased interaction degree between the DMN and CEN in MDD.

### 4.1. Aberrance of Connectivity Degree in Nodes

Increased degree of rAI is found in MDD, which means the interactivity between the rAI and the other brain regions increases in MDD during resting state. This suggested that the aberrance of rAI contributes to the cognitive impairment of depression in rest. The deficit of rAI was found in the literature in MDD [13]. Meanwhile, some researches have proved that rAI, which is an important brain region of SN [7], is associated with neural cognition, interoception, affection process, and subjective and autonomic function [25], which are all associated with depression [5]. The aberrance of rAI contributes to the dysfunction switch of the DMN and CEN in resting state [13, 26]. The disorder of the connectivity degree of rAI between other regions in the triple network model suggested that more activity is needed to keep the normal regulation in the MDD.

### 4.2. Aberrance of Interregional Connectivity

The alteration of interregional connectivity between the rAI and the other regions in the triple networks was detected. The interregional connectivity *η* increases occurred in the vmPFC, rdlPFC, ldlPFC, rPPC, lPPC, PCC, rAng, and lAng brain regions. All these increasing *η* contributed to the significantly increased connectivity degree of the rAI and further proved the important role of the AI in MDD. The other brain regions are all important for the neurocognition, such as the research showing that the brain regions of PCC and Ang are related to the episodic memory retrieval [27], autobiographical memory [28], and semantic memory related to internal thought [29]. The vmPFC is associated with self-related and social cognitive processes [30], value-based decision making [31], and emotion regulation [32]. The decreased function of dlPFC in MDD has been detected in resting [33], which matches the result of our study shown in Figure 2. Though the CEN is composed of portion of prefrontal lobe and parietal lobe, a lot of investigations show the main abnormal functional connection of several psychiatric disorders associated with the aberrance of dlPFC, including the depression [4, 34, 35].

### 4.3. Aberrance of Networks

For MDD, the functional connectivity degree between the DMN and CEN significantly decreases compared with the HC in the current study. The network DMN is involved in self-referential oriented process, which is active in the resting state [36] and is deactivated during goal-directed tasks [9]. The CEN is responsible for the high-level external cognitive tasks and modulation of mood reaction [37] in both of resting state [34] and stimulus task [35] in major depression. The investigations have found dysfunction in both of the two cognitive networks in MDD [38]. The results suggested the decreased functional interaction between the DMN and CEN of the MDD in the resting state. Our findings couple with the statement of the aberrant network connectivity in major depression [10, 39]. The results might suggest that the patients with MDD cannot normally regulate the switching between the internal self-reference, working memory, autobiography, decision making and the external stimulus, orientation tasks, and demand cognitive action in rest.

According to the triple model proposed by Menon [4], it is mentioned that SN has a core role in mediating the conversion of the functional connectivity between the DMN, which is related to the self-referential cognition [36], and CEN, which is related to the external oriented task [37], especially the region rAI which is part of SN [4, 26]. A lot of researches demonstrate that the rAI occupies an important position in the interaction between the DMN and CEN in the HC [26] and MDD [8] and also in other psychogenic disorders. Recently, Manoliu et al. found that the aberrance of the rAI may be associated with the disorder interaction between DMN and CEN in schizophrenia [2, 12] and major depression [13]. In our study, patients with MDD could not well modulate the dynamic interaction between the DMN and CEN. All of these researches provided evidence for the assumption of the triple network model, which might be a common frame for understanding dysfunction in the three core networks of variety in cognitive disorders and supported the proposal of the triple network model. It showed the contribution of the rAI to the depression and suggested a link between the MDD and the dysfunction interaction of the DMN and CEN.

## 5. Conclusion

The results showed that increased connectivity degree occurred in right anterior insula (rAI) in MDD compared with HC and decreased connectivity degree between DMN and CEN in MDD. These abnormalities may indicate that the functional connectivity increased between the SN and CEN for MDD and the dynamic interaction between the DMN and CEN decreased for MDD. All the results provided new insights into our understanding of depression.

## Figures and Tables

**Table 1:** Demographic and clinical characteristics.

Variables (mean ± SD)	MDD (*n* = 16)	HC (*n* = 16)	*P* value^a^
Gender (M : F)	4 : 12	4 : 12	1
Age (years)	33.13 ± 8.29	39.13 ± 10.22	0.10
Education level (years)	13.75 ± 3.01	12.93 ± 2.40	0.61
Age range	21–57	21–55	
Duration of illness (years)	7.66 ± 8.29	—	
Number of depressive episodes	2.63 ± 1.26	—	
HAMD	21.44 ± 3.97	—	
HAMA	16.00 ± 9.61	—	

MDD: major depressions disorder; HC: healthy controls; SD: standard deviation; HAMD: Hamilton Depression Rating Scale.

^a^ *P* value for the two-sample *t*-test of MDD and HC.

**Table 2:** Spatial coordinates of the nodes among the triple networks.

Network	Brain region	BA	Coordinates (MNI)			*T*
			*x*	*y*	*z*	
DMN	lAng	39	−48	−67	34	8.94
	rAng	39	54	−61	30	5.58
	PCC	30	0	−52	18	13.90
	vmPFC	10	−1	56	10	5.05

CEN	lPPC	7	−33	−70	50	7.73
	rPPC	7	42	−62	50	7.56
	ldlPFC	6	−27	20	58	6.03
	rdlPFC	6	27	20	62	3.60

SN	dACC	32	2	34	25	3.27
	lAI	45	−34	23	4	4.46
	rAI	45	34	23	5	3.50

BA: Brodmann areas; MNI: Montreal Neurological Institute spatial array coordinates; *T*: *t*-value.

**Table 3:** The connectivity degree between networks and the *P* value by two-sample *t*-test.

Variables (mean ± SD)	MDD	HC	*P* value (MDD versus HC)
DMN-CEN	7.15 ± 2.72	9.27 ± 1.57	0.01^*^
DMN-SN	1.63 ± 3.16	0.73 ± 2.72	0.40
CEN-SN	2.70 ± 2.87	2.19 ± 2.74	0.61

^*^Significant by two-sample *t*-test.
